# Effect of calcium oxalate microcrystals on kidney proximal tubule epithelial cell gene expression in microgravity

**DOI:** 10.21203/rs.3.rs-6883199/v1

**Published:** 2025-06-27

**Authors:** Kendan Jones-Isaac, Catherine K. Yeung, Jacelyn Bain, Kevin Lidberg, Jade Yang, Lu Wang, James MacDonald, Theo Bammler, Kenneth E. Thummel, Megan Corn, Micaela Vela Ruiz, Stefanie Countryman, Paul Koenig, Jonathan Himmelfarb, Edward J. Kelly

**Affiliations:** University of Washington; University of Washington; University of Washington; University of Washington; University of Washington; Sciences University of Washington; Sciences University of Washington; Sciences University of Washington; University of Washington; University of Washington; University of Washington; University of Colorado; University of Colorado; Mt Sinai School of Medicine; University of Washington

## Abstract

We evaluated the impact of spaceflight on a microphysiologic model of calcium oxalate (CaOx) kidney stone disease. Proximal tubule epithelial cells cultured as confluent microtubules were exposed to CaOx crystals with or without potassium citrate (a potential countermeasure) to determine impact on gene expression. Nine genes were differentially expressed in response to CaOx crystal exposure during spaceflight. This project presents the use of microgravity as a unique environment to study kidney pathophysiology.

## Introduction

Almost 12% of the global population is affected by kidney stone disease (KSD) with the experience being reported as painful and debilitating. Estimated recurrence rates for KSD have been reported as high as 67%— further compounding the considerable disease burden on the individual patient and economic strain on healthcare systems globally^1^. KSD poses an even greater threat in microgravity due to the inaccessibility of medical and surgical interventions readily available on earth. Exposure to microgravity increases the risk of kidney stone disease in humans^2^ due to a dehydration-like state caused by altered body fluid distribution, accelerated bone and muscle atrophy, and oxidative stress responses to elevated ROS generation during spaceflight^2^, increased inflammation and oxidative stress response^3^, and a diet enriched in protein and sodium^2,4^. A recent report confirmed the potential for spaceflight-induced kidney dysfunction, highlighting renal transporter dephosphorylation, expansion of the distal convoluted tubule, loss of tubule density, and galactic cosmic radiation-caused kidney impairment in human datasets and spaceflight exposed rodents^5^.

Citrate supplementation in the form of potassium citrate (KCit), an inexpensive and well-tolerated medication, has been used as a pharmacologic countermeasure to decrease the risk of kidney stone formation by reducing hypercalciuria in both in spaceflight crew and the general public^4^. Prevention of symptomatic kidney stone formation is critical as larger stones require surgical intervention including extracorporeal shock wave lithotripsy to pulverize stones or insertion of instrumentation percutaneously or via the urethra to mechanically extract stones from the kidney to prevent irreversible kidney damage^6^.

Flow-mediated 3D cultures of proximal tubule epithelium, referred to as kidney microphysiological systems (MPS), can be used to study kidney function and biomarkers of developing pathologies^7^. Various stressors, including environmental toxins, medications, excess heat, and particulates can be introduced into the system to elicit physiological responses in the human kidney^8^.

**Figure 1:**
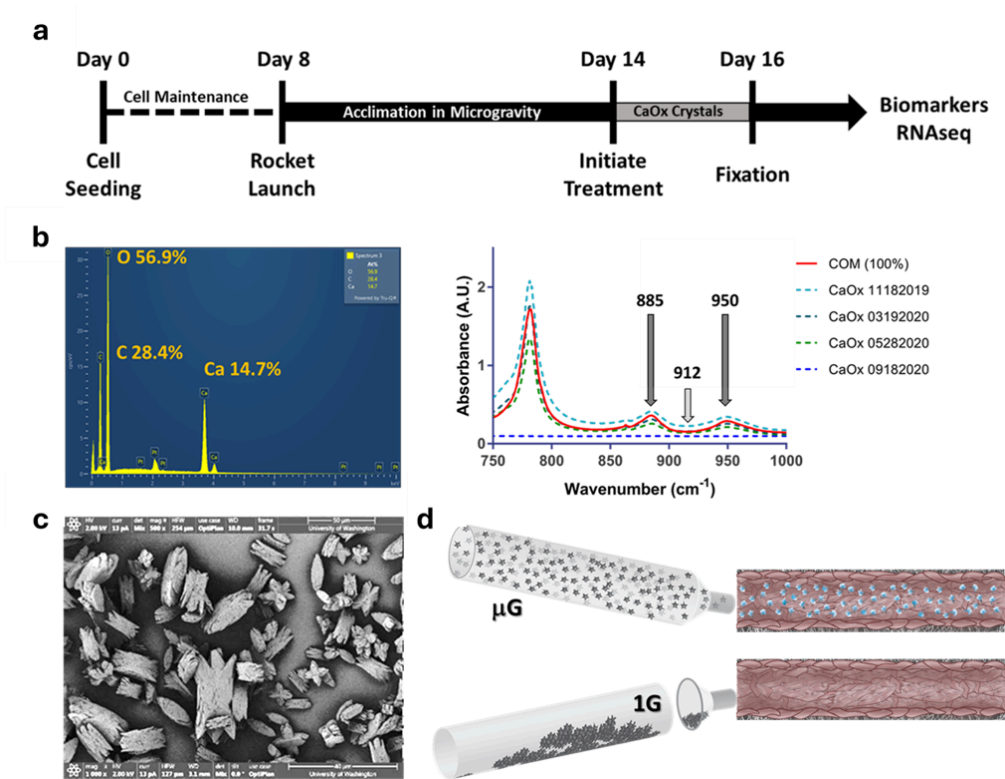
Summary of Kidney Cells-02 experiment. (a) Experimental timeline for KC-02 (b) energy-dispersive (L) and Fourier-transform infrared (R) spectrographs of lab generated COM microcrystals, (c) SEM of lab generated COM microcrystals, (d) microgravity facilitates PTEC exposure to COM while crystal density inhibits exposure at 1G.

To study KSD pathophysiology, exposure to microcrystals (approximately 10 microns) of calcium oxalate monohydrate (COM), precursors of developing kidney stones, can be used to model early events underlying kidney stone disease progression. Biomarkers of kidney injury released into the effluent in response to COM crystal exposure can indicate perturbed signaling/stress response pathways and may serve to guide disease treatment options. For example, interleukin 6 (IL-6), a cytokine marker of chronic inflammation, is elevated in vivo and in kidney MPS after exposure to known nephrotoxins^9,10^. However, MPS experiments with microcrystal challenge cannot be conducted in ground-based laboratories due to gravitational settling of the microcrystals in media reservoirs, thus preventing introduction of the COM microcrystals into the MPS at physiological rates of flow and fluid shear stress (Fig. 1d). In the unique microgravity environment of the International Space Station National Laboratory (ISSNL), COM microcrystals are evenly dispersed in the treatment media allowing access to the proximal tubule epithelial cells lining the internal culture chamber within the kidney MPS to induce a physiological response to COM exposure. Investigating the cellular response to COM challenge in microgravity can provide insight into biochemical pathways of stone response in the kidney proximal tubule in microgravity and interrogate the mechanism of potassium citrate efficacy.

## Results

### COM Crystals.

Calcium oxalate monohydrate crystals were generated with more than 95% purity. Scanning electron microscopy imaging of COM preparations was consistent with those found in literature showing primarily sharp edges and greater relative surface area than comparable dihydrate crystals^11^. Energy-dispersive X-ray spectra from these preparations showed the characteristic Ca:C:O ratio of 1:2:4 confirming the identity as CaOx. FTIR peaks at 950 cm-1 and 885 cm-1 confirmed the presence of the monohydrate (Fig. 1b). Mean crystal size was (mean ± std dev): length: 15.8 ± 5.7 μm and width: 8.9 ± 4.0 μm as determined by scanning electron microscopy (Fig. 1c).

### Onboard operations.

Operations on the ISSNL (CRS-22) were nominal with expected high performance from the hardware and crew. In comparison with previous flight experiments (CRS-17)^12^ minimal fungal contamination was observed and we were able to isolate high quality RNA (RIN > 7) from 70 out of 72 tubules (97.2%). 72% of tubules exhibited nominal media flow volumes indicating that the tubules were under constant perfusion (**Supplemental Table 2**).

### IL-6 effluent concentration in control tubules (flight vs. ground).

IL-6 levels in effluents collected from control media showed a significant increase during spaceflight of 71% (p < 0.0001) **(Supplementary Fig. 1)** compared with ground controls.

### RNAseq global transcriptomics (Control, COM, KCit).

**Figure 2:**
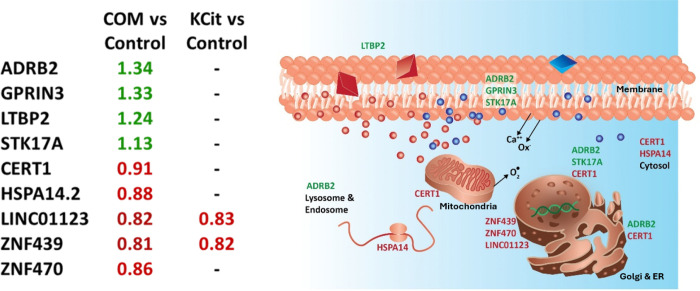
Genes differentially expressed by MPS cultured PTECs in response to COM crystal treatment during spaceflight. (a) Nine genes were differentially expressed in COM treated cells vs. control media (left column) or COM treated cells vs. COM + KCit treated cells (right column). Values indicate observed fold change with upregulated genes in green and downregulated genes in red. (b) Schematic of subcellular location of differentially expressed genes.

Nine genes were significantly differentially expressed in response to COM crystals vs control media in flight; four were upregulated and five downregulated. The four upregulated genes in response to COM treatment in flight, ADRB2, GPRIN3, LTBP2 and STK17A were increased 1.34-fold, 1.33-fold, 1.24-fold, and 1.13-fold, respectively. Treatment with COM in flight led to the downregulation of COL4A3BP (0.91-fold), HSPA14 (0.88-fold), ZNF439 (0.81-fold) and ZNF 470 (0.86-fold) and one non-protein encoding RNA, LINC01123 (0.82-fold). The addition of KCit reversed the impact of COM treatment on seven of the nine genes. Only LINC01123 and ZNF439 remained insensitive to the effects of KCit. In both cases, the genes remained downregulated to the same extent as with COM treatment alone. (Fig. 2). Flight vs. ground comparisons were of limited value as on earth, COM microcrystals settled in the media reservoir and were not delivered to the cell culture chamber, thus the data was not included in this analysis.

## Discussion

The risk of kidney stone formation during space travel is known to increase due to systemic factors including dehydration, increased bone resorption, oxidative stress, dietary factors,^2^ and kidney specific effects including renal transporter dephosphorylation, expansion of the distal convoluted tubule, and loss of tubule density^5^. However, little is known regarding the effects of microgravity on the microenvironment of the kidney proximal tubule. Evaluating the proximal tubule epithelium response to calcium oxalate microcrystal precursors is facilitated by the microgravity environment and allows the assessment of simultaneous exposure to microgravity and oxalate microcrystals. We report herein the second successful deployment of the Kidney Chip Perfusion Platform (KCPP) hardware system^13^ to test the hypothesis that PTEC gene expression is modified when exposed to oxalate microcrystals under microgravity conditions. Specifically, the KCPP delivered control or treatment (1000 μg ml^−1^ COM suspension) media to 24 kidney chips (72 independent tubules) on orbit and corresponding number of ground-based control chips. Study results show both increased and decreased cell gene expression when exposed to oxalate microcrystals under microgravity conditions, effects that were largely reversed by co-treatment with KCit. We explored the RNA transcriptomic effects of KCit as a countermeasure to modulate the effects of COM exposure, observing that co-infusion of KCit along with COM normalized the transcription of 7 of the 9 differentially expressed transcripts (with the exception of ZNF429 and LINC01123). While the roles of the differentially expressed transcripts in kidney stone disease are difficult to pinpoint, many of the DEGs are involved in calcium regulation, RNA synthesis, epithelial cell homeostasis, and stress response (**Supplementary Table 4**). Further study is required to determine if these transcripts are useful in designing treatments to prevent or treat kidney stones.

In ground-based laboratories, attempts to deliver COM to the tubules in the kidney chip were unsuccessful due to gravitational settling of the microcrystals in the input media reservoirs or syringe pumps. Thus, we believe that the PTEC-MPS system, in microgravity, represents a significant improvement in the ability to study the intratubular response to oxalate microcrystals and may help to identify sentinel events or predisposing factors (e.g., inflammation, oxidative stress, genetic background, disease history) that increase risk of pathologic stone aggregation both in space and on earth.

This study is limited by the short exposure to microgravity (8 days) and a small sample size. Recently published work describing multi-omic profiling of the SpaceX Inspiration4 civilian crew showed that significant changes occurred in physiological and stress responses after 3 days of spaceflight, some of which parallel long-term microgravity residence^14^. A future goal includes maintaining the PTEC-MPS for extended (~ 6 months) exposure to microgravity to determine if long-term exposure results in cumulative loss of function, or possibly activation of cellular adaptation mechanisms that reverse the changes observed in short term spaceflight.

While the cell donors for this experiment were selected from diverse sex and racial backgrounds, we were unable to identify donors with a known history of kidney stone disease. We hypothesize that the genetic background of a donor may affect the PTEC response to stone precursors, predisposing the individual to obstructive stone formation. Future experiments should include donors with a history of kidney stone disease, as well as genetic polymorphisms (e.g., CASR, SLC34A1, and CYP24A1)^15^ that have been associated with kidney stones in a recent GWAS study.

Taken together, the findings of this study suggest that the PTEC-MPS in a microgravity environment is a useful system to evaluate early intratubular pathophysiological changes. These changes may uncover novel therapeutic targets that can be used in the development of pharmacologic agents to maintain the health of not only space explorers but also the general public.

## Methods

### Synthesis of calcium oxalate crystals.

Calcium oxalate (CaOx) crystals were generated from 10 mM stocks of calcium chloride (CaCl_2_) (Fisher Scientific, NH, USA) and sodium oxalate (Na_2_C_2_O_4_) (Fisher Scientific, NH, USA) in sterile H_2_O further diluted to 2 mM working solutions. These solutions were combined in a 2 L Erlenmeyer flask at a volume of 500 mL each in a 1:1 ratio and incubated for 24 h at room temperature. To harvest the crystals, the solution was aliquoted into 50 mL conical tubes and centrifuged for 5 minutes at 3000 rpm. The supernatant was then aspirated until only the crystals, and a small amount of supernatant was present. The remaining fluid and crystals were combined into a 15 mL conical tube, and the solution was centrifuged again for 5 min at 3000 rpm. The solution was then aspirated down to the crystalline pellet. 1 mL methanol (MeOH) was then added to the crystal sample and transferred to a weighed Eppendorf tube. The crystals were spun in a microcentrifuge for 5 min, the MeOH was aspirated, and the mass of the Eppendorf with the crystals was determined. The crystals were then left to dry overnight at 37° C. To recrystallize the sample, calcium chloride and sodium oxalate were dissolved in sterile water at a concentration of 10 mM each, heated at 37° C for 1 h and then cooled overnight at 4° C. The resulting microcrystals were then sterilized by the addition of 1 mL of MeOH. The crystals were then spun in a microcentrifuge and left to dry overnight at 37° C and collected into a sterile tube.

### Characterization of calcium oxalate monohydrate (COM) crystals.

Fourier-Transform Infrared Spectroscopy (FTIR), Scanning Electron Microscopy (SEM), and Energy-Dispersive Spectroscopy (EDS) were used to characterize the COM crystals. To create the disc for FTIR analysis, 2 mg of COM crystals and 200 mg of KBr were ground with an agate mortar and pestle until smooth. The powdered mix was then poured into a die and compressed with a pneumatic press for 5 minutes at a pressure of 15000–16000 psi with a vacuum hose attached to remove any excess water. The disc containing the COM crystals was placed in a PerkinElmer^®^ (Shelton, CT) Frontier FTIR spectrometer and read at 16 scans, 0.5 cm^−1^ resolution and the range of 500–4000 cm^−1^. SEM of prepared COM crystals were analyzed using a FEI (Hillsboro, OR) XL830 dual beam SEM-FIB; EDS was performed using a ThermoFisher Scientific Apreo connected to MAPS3 software.

### Generation of Kidney Microphysiological Systems and Culturing in Kidney Chip Perfusion Platform.

Demographics for the donors used in this experiment can be found in **Supplementary Table 1**. Methods for maintenance, treatment and fixation of the kidney MPS devices are detailed previously^16^. In brief, proximal tubule epithelial cells were isolated from whole human donor kidneys (not suitable for transplantation) obtained from Novabiosis (Durham, NC), cryopreserved, and commercially shipped on dry ice to Kennedy Space Center (KSC) where they were cultured and seeded onto 10–15 triplex (TSC) devices per donor to achieve ≥8 fully confluent TSCs per donor. The TSC devices were maintained within the pneumatic Kidney Chip Perfusion Platform system (KCPP) at a flow rate of 1 μL min^−1^. The integration and operation of the KCPP has been described previously^13,16^. Cultures were incubated for 3–7 days in a standard laboratory incubator prior to transfer to KCPP. Kidney MPS devices were subjected to 1 g (control) or microgravity conditions (CRS-22, KSC launch 17:29 UTC, 06/03/21 and ISSNL docking at 09:09 UTC, 06/05/21) for 7 days before media exchange to control (unsupplemented media) or treatment media containing either 1000 μg ml^−1^ COM crystals or 1000 μg ml^−1^ COM crystals + 250 μm KCit (potassium citrate tribasic monohydrate, Sigma, St. Louis, MO). The cassettes containing control or treatment media were inverted 4 times to evenly distribute COM microcrystals prior to initiating flow. Exposure to COM crystals and KCit in the ground-based laboratories at KSC or at the ISSNL was performed as previously described^12^. Upon completion of the test phase, the KCPP was flushed with RNAlater at a flow rate of 10 μL min^−1^ for 2 h before being placed in ultracold stowage (ISSNL) or −80° C freezer (KSC). ISSNL samples were returned to earth (undocking at 14:45 UTC, 08/08/21) and shipped to the laboratory at UW.

### RNA Collection from PT-MPS Devices.

Collection of RNA samples from PT-MPS tubules, RNA extraction and RNAseq analysis were performed as previously described^17^. In brief, RNA was collected by injecting 1000 μl of buffer RLT (Qiagen, 79216) and collecting the lysate from at the outflow port in sterile Eppendorf tubes. The lysates were then stored at − 80° C and shipped on dry ice to Novogene for RNA isolation and analysis (Sacramento, CA). Following RNA purification, integrity was assessed by Bioanalyzer (Agilent, Santa Clara, CA). All samples with RIN scores > 7 were used for library synthesis and RNAseq analysis as previously described^17^. Gene ontology analysis was performed using the Comparative Toxicogenomics Database^18^.

## Supplementary Material

Supplementary Files

This is a list of supplementary files associated with this preprint. Click to download.

SupplementaryFiguretables.docx

## Data Availability

The datasets generated and/or analyzed during the current study are available upon reasonable request from the corresponding author.
